# The Role of Oxytocin and Vasopressin in Drug-Induced Reward—Implications for Social and Non-Social Factors

**DOI:** 10.3390/biom13030405

**Published:** 2023-02-21

**Authors:** Olga Wronikowska-Denysiuk, Weronika Mrozek, Barbara Budzyńska

**Affiliations:** Independent Laboratory of Behavioral Studies, Chair of Biomedical Sciences, Medical University of Lublin, Chodzki 4a Street, 20-093 Lublin, Poland

**Keywords:** addiction, drug abuse, oxytocin, vasopressin, social behavior, peptides

## Abstract

Drug abuse is a worldwide problem that leads to negative physical, mental, and economic consequences. Although pharmacological strategies for drug addiction management have been widely studied, therapeutic options with high efficacy and a low side-effects profile are still limited. Recently, there has been a growing interest in oxytocin (OT) and vasopressin (AVP) systems as potential therapeutic targets for the treatment of drug abuse. OT and AVP are hypothalamic neuropeptides involved in numerous physiological processes. Additionally, studies show that these neurohormones are highly implicated in the modulation of a wide range of behaviors. Interestingly, ample evidence has shown that both, OT and AVP are able to decrease the consumption of different drugs of abuse, as well as to ameliorate their rewarding and reinforcing effects. Furthermore, OT and AVP have been strongly involved in prosocial effects and social reward. In particular, OT has been shown to be able to shift drug-induced reward into social-induced reward, mainly due to its interaction with the dopaminergic system. This phenomenon is also reflected in the results of clinical trials where intranasal OT shows promising efficacy in managing substance use disorder. Therefore, the aim of this review is to comprehensively characterize the involvement of OT and AVP in the rewarding and other behavioral effects of drugs of abuse in animal models, with a particular highlight on the impact of social factors on the observed effects. Understanding this relationship may contribute to higher drug development success rates, as a result of a more profound and deliberate studies design.

## 1. Introduction

According to the latest European Monitoring Centre for Drugs and Drug Addiction (EMCDDA) report, around 83 million or 28.9% of adults (aged 15–64) in the European Union are estimated to have used illicit drugs at least once in their lifetime. The most prominent emerging challenges presented by the European drug market consist of the widespread availability of a diverse range of drugs, their increasingly high purity or potency, and importantly, the ever more frequently observed transition from experimental to habitual and dependent consumption [1]. In the light of such circumstances, the definition of vulnerability factors that may influence the liability to drug abuse is a vastly important issue.

One of the factors that can impact the intensity of the rewarding experience derived from the intake of illicit drugs is social context during their consumption. Numerous studies in animal models [2,3,4,5], including our own study [6], have shown that drug-induced reward can be highly dependent on the social context, e.g., the company of the conspecific during drug intake. A similar pattern has been reported in humans where the social environment in which the drug is taken has influenced the liability to its abuse [7,8]. Furthermore, other social-dependent factors such as paternal behavior or friendships with peers have been shown to influence drug abuse and the development of drug addiction [9,10,11,12].

Many overlapping mechanisms can be responsible for the social-dependent and social-independent changes in drugs-triggered effects. Nevertheless, oxytocin (OT) and arginine vasopressin (AVP) are two neurohormones considered as important players in drug-related reward [3,13,14]. OT and AVP are the hypothalamic neurohormones, released into the bloodstream by the posterior pituitary [15]. The primary role of OT consists in the regulation of reproductive processes, and stimulation of labor and breast milk production [15,16], whereas the primary AVP function involves regulation of blood pressure by kidney- and vessel-associated mechanisms [15,17]. Nevertheless, these neurohormones additionally trigger complex effects in the human body, and thus, they have been appreciated in addition as neuromodulators capable of regulating social cognition and affecting a wide range of behaviors, such as social attachment, social exploration, recognition and aggression, pair bonding, as well as maternal, affiliative and sexual behaviors [18,19]. Furthermore, recent studies in humans have also suggested that central OT modulates social cognition, including an increase in interpersonal trust, eye gaze, face recognition, and the ability to infer the emotions of others based on facial cues [20].

Recently, there has been a growing interest in OT and AVP systems as potential therapeutic targets for the treatment of drug abuse. Therefore, the aim of this review is to comprehensively characterize the involvement of OT and AVP in the rewarding and other behavioral effects of drugs of abuse in animal models, with a special highlight on the impact of social factors on the observed effects. Specifically, the relationship between OT and AVP neurotransmission and the behavioral/rewarding effects of 3,4-methylenedioxymethamphetamine (MDMA), cocaine, ethanol, amphetamine, methamphetamine (METH), morphine, heroin and nicotine was reviewed in this paper. Because several clinical trials on OT use in drug addiction have already been conducted (see Section 6), a thoughtful insight into ligands acting via receptors for OT and AVP may result in identifying novel therapeutic targets for the treatment of substance use disorder.

## 2. Neuromodulation of OT and AVP

**Figure 1:**
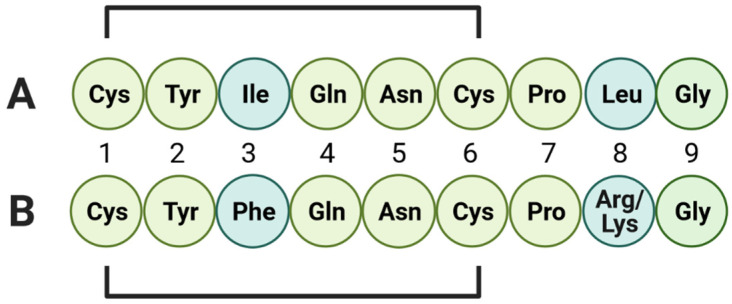
The structure and amino acids sequence of (**A**) OT and (**B**) AVP. The lines indicate cysteine residues that form a sulfur bridge by disulfide bonds. OT and AVP structures differ in the first and eighth place in the aminoacid sequence (dark green). This figure was created with https://biorender.com/ (accessed on 10 January 2023).

OT and AVP are neuropeptides consisting of nine aminoacids (nonapeptides) and differing only in two of them, at position 3 and 8 (Figure 1).

There are two major hypothalamic cells responsible for OT and AVP synthesis: magnocellular neurons and parvocellular neurons [21]. Previous theories assumed that these neurons were responsible for transmitting different signals. Precisely, it was believed that magnocellular neurons project to the posterior pituitary gland from where OT and AVP are released into the bloodstream, whereas parvocellular neurons project to other parts of the brain regulating different types of behavior. Nevertheless, the newest data, extensively reviewed by Grinevich and Ludwig (2021) [22], suggest that magnocellular OT and AVP neurons may additionally send axonal collaterals to forebrain regions and release these neuropeptides in micro volumes non-synaptically to modulate and control various behavioral responses (additionally by producing releasing factors, as it is reported for classical hypothalamic neurons) [23,24,25]. Importantly, the magnocellular OT neurons are controlled by parvocellular OT neurons in order to maintain homeostasis and regulate behavioral response. Nevertheless, a similar pattern has not been observed for parvocellular AVP neurons, and, at this stage of knowledge, they cannot be considered as independent cells [22].

The location of OT neurons and AVP neurons in rodents’ brains is similar; however, some differences have been identified. OT neurons are expressed mainly in the hypothalamic paraventricular nucleus (PVN) and supraoptic nucleus (SON) with an additional minor scatter in the bed nucleus of stria terminalis (BNST) [19,22,26]. AVP neurons are also expressed in PVN and SON but have been found additionally in the suprachiasmatic nucleus (SCN), BNST, medial nucleus of the amygdala and entorhinal cortex [27,28]. Interestingly, OT and AVP neurons can project to distinct locations through the brain, including the obvious, the pituitary, but also other regions and structures such as the amygdala, hippocampus, striatum, brainstem and spinal cord [19,22,29].

The effects of OT and AVP are exerted via modulation of their receptors (OT receptors, OTRs and AVP receptors AVPRs, respectively). These are G-protein coupled receptors (GPCR) and their activation leads to a conformational change in the receptor structure which leads to the activation of G proteins and subsequent Ca^2+^ release from intracellular stores [30,31]. One receptor for OT (OTR) and three types of AVPRs (AVPV_1A_Rs, AVPV_1B_Rs and AVPV_2_Rs) have been identified [32,33,34]. Extensive studies on rodents have identified the sites of expression of OTRs and AVPRs, both centrally and peripherally. In the brain, OTRs are expressed, among others, in the amygdala, nucleus accumbens (NAc), BNST, PVN, medial preoptic area, ventromedial nucleus of the hypothalamus, hippocampus, ventral pallidum, periaqueductal gray, striatum, lateral septum, ventral tegmental area, and olfactory bulb. OTRs are additionally expressed peripherally, mainly in the uterus, placenta and cardiovascular system (for a detailed list of central and peripheral OTRs locations see [29,33]). AVPV_1A_Rs are expressed both peripherally, largely on the vascular smooth muscle but also in the testis, uterus, liver, blood vessels, and renal medulla, as well as centrally, in different brain areas, such as the lateral septal nucleus, thalamic nuclei, hippocampus, parts of the basal ganglia, and different brainstem nuclei [35,36]. AVPV_1B_Rs are detectable mainly in the anterior pituitary, whereas AVPV_2_Rs are found mainly in the kidneys [29].

OT is released in response to sexual stimulation, stretching of the cervix and uterine dilatation during labor and with stimulation of the nipples during breastfeeding [15]. AVP displays a binary effect and is released into the circulation in response to extracellular fluid hyperosmolarity with a subsequent (1) increase in the reabsorption of solute-free water to blood from kidneys’ filtrate and an (2) increase in peripheral vascular resistance triggered by vessels constriction [15,17]. Apart from OT and AVP systemic activity, ample evidence has proven their involvement in regulating social and aggressive behaviors, stress adaptation, social memory, as well as maternal and sexual behaviors [37].

## 3. The Impact of OT/AVP and OTRs/AVPRs Ligands on Behavioral and Rewarding Effects of Drugs of Abuse in Animal Models

**Figure 2:**
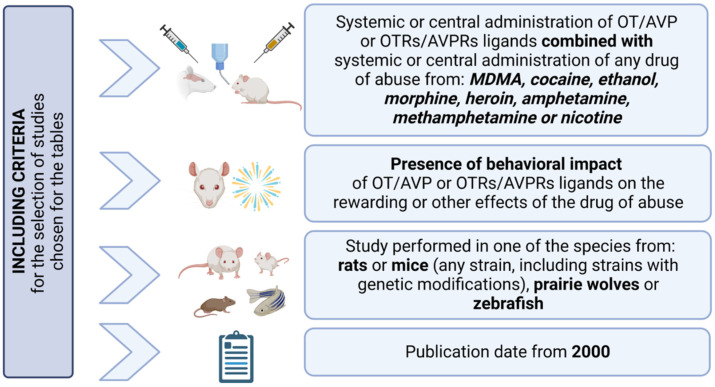
Including criteria for the selection of studies chosen for the Table 1, Table 2, Table 3 and Table 4. This figure was created with https://biorender.com/ (accessed on 10 January 2023).

Extensive studies in animal models have proven the involvement of OT and AVP neurotransmission in behavioral and rewarding effects of different drugs of abuse. Specifically, the effects of OT, OTRs ligands, AVP and AVPRs ligands have been summarized in this review. The selection of studies included in these tables has been based on the following criteria: (1) systemic or central administration of OT/AVP or OTRs/AVPRs ligands combined with the systemic or central administration of any drug of abuse from MDMA, cocaine, ethanol, morphine, heroin, amphetamine, METH or nicotine; (2) presence of the behavioral impact of OT/AVP or OTRs/AVPRs ligands on the rewarding or other effects of the drug of abuse; (3) study performed in one of the species from rats or mice (any strain, including strains with genetic modifications), prairie wolves or zebrafish; (4) publication dates from 2000 (Figure 2). The choice of the animal species included in this review has been based on (a) their sociability and willingness to live in groups and (b) the presence of data on the chosen drugs of abuse and correlated addictive-like behaviors in those species. The abundance of presented data certainly proves the involvement of OT/AVP in the modulation of different effects of drugs of abuse; nevertheless, to comprehensively summarize collected data, several aspects require underlining, clarification and comment.

### 3.1. The Involvement of OT Transmission in Drug-Induced Reward

Findings reported in Table 1 and Table 2 suggest a general conclusion that the administration of (1) OT itself; (2) OT analog (Thr4,Gly7-OT) or (3) OTRs agonists (PF-06655075, WAY-267464 or carbetocin) and subsequent activation of OT transmission lead to attenuation of the rewarding effects of different drugs of abuse.

Such effects have been observed in different behavioral paradigms where OT has been shown to decrease the rewarding effects of METH [38,39] and morphine [40], measured in CPP. Furthermore, OT has been proven to reduce the intake of cocaine [41,42], METH [43,44,45] and ethanol [46,47,48,49,50,51,52,53,54]. Additionally, OT prevented the drug-, stress- and cue-induced reinstatement of cocaine [41,55,56,57,58,59,60], METH [39,44,45,61,62,63,64,65,66,67,68,69,70,71,72] and ethanol [73,74]. Finally, OT has been shown to prevent seeking during extinction and to facilitate the extinction of cocaine- [58,59] and METH-addictive effects [39].

Similar effects were observed for agonists of OTRs which were also shown to ameliorate drug reward. For example, carbetocin was able to prevent stress- and morphine-induced reinstatement to morphine-seeking [75,76] and attenuate the acquisition, facilitate extinction and block reinstatement of ethanol-induced CPP [77]. OT analog, Thr4,Gly7-oxytocin was able to block the cue-induced reinstatement of cocaine seeking [60] and the OTRs agonist while PF-06655075 was shown to decrease ethanol intake in dependent rats [52].

Interestingly, one study performed in adolescent rats reported that peripheral OT administration led to an increase in nicotine intake measured in two bottle free-choice paradigms [78], which could suggest that OT effects on drug-induced rewards may be age-dependent. Nevertheless, another study that took age into account showed that adolescent pretreatment with OT reduced METH-self administration and reinstatement to METH abuse in adult rats [44]. Therefore, the interaction between OT and the age-dependent effects of a drugs-induced reward needs further clarification. Importantly, the attenuation of drug reward triggered by OT activation can be prevented by the administration of OTRs antagonists (L,368,899 and atosiban) [39,47,72,79], which proves the involvement of OTRs in the observed effects. The effects of OT and OTRs ligands on behavioral/rewarding effects of different drugs of abuse has been summarized in Table 1 and Table 2, respectively. 

**Table 1:** The effects of OT on behavioral/rewarding effects of different drugs of abuse.

Species and Sex	Drug of Abuse and Dose	OT Effective Dose	Behavioral Test	OT-Induced Effect	Ref.
Long–Evans rats ♂	MDMA 2.5 mg/kg, ip	0.25 mg/kg, ip	SI	↑ in adjacent lying	[80]
Sprague Dawley rats ♂, ♀	cocaine (♂) 0.2 mg/50 μL/bolus, iv (♀) 0.15 mg/50 μL/bolus, iv	0.1, 0.3, 1, 3 mg/kg, ip	SA	↓ cocaine intake (in ♀)	[41]
		1.0 mg/kg, ip	SA	↓ cue-induced cocaine seeking following extinction (in ♀)	
Sprague Dawley rats ♂, ♀	cocaine 0.5 mg/kg/infusion, iv	0.6 nmol/0.25 μL/side into NAc	SA	↓ cue-induced reinstatement of cocaine seeking	[56]
		0.6 nmol/0.25 μL/side into PFC	SA	↑ reinstatement to cocaine-associated cues	
Sprague Dawley rats ♂	cocaine 0.2 mg/50 μL/infusion, iv 10 mg/kg, ip, for cocaine- priming	0.3, 1, 3 mg/kg, ip during SA	SA	↓ cocaine intake	[42]
		0.3, 1 mg/kg, ip during reinstatement	SA	↓ cocaine prime-induced (0.3 and 1 mg/kg) and cue-induced (1 mg/kg) reinstatement to cocaine seeking	
Sprague Dawley rats ♂, ♀	cocaine (♂) 0.2 mg/50 μL/infusion, iv (♀) 0.16 mg/50 μL/infusion, iv	0.3, 1 mg/kg, ip	SA	↓ cocaine-seeking during extinction and cue-induced reinstatement of cocaine-seeking	[58]
Sprague Dawley rats ♂, ♀	cocaine (♂) 0.2 mg/50 μL/bolus, iv (♀) 0.15 mg /50 μL/bolus, iv	1 mg/kg, ip	SA	↓ cue-induced cocaine reinstatement	[55]
		3 μg/0.5 μL/side, icv	SA		
OF1 mice ♂	cocaine 1, 10 mg/kg, ip	1 mg/kg, ip	CPP	↓ social defeat-induced increase of cocaine (1 mg/kg) rewarding effects; facilitation of the extinction of cocaine (10 mg/kg)-CPP;	[59]
	cocaine 0.5 mg/kg/infusion, iv 10 mg/kg, ip for cocaine-priming		SA	facilitation of the extinction of cocaine-seeking behavior; ↓ of the cocaine-primed reinstatement of social defeat-induced cocaine-seeking	
Sprague Dawley rats ♂	cocaine 0.25 mg/0.1 mL.infusion, iv	10 ng/10 μL, icv	SA	↓ reinstatement of cue-induced cocaine seeking behavior	[60]
			EPM	↓ anxiety triggered by cue-induced reinstatement conditions and cocaine-paired conditioned locomotion	
Sprague Dawley rats ♂, ♀	METH 0.1 mg/kg/50 μL/infusion, iv	0.3, 1 mg/kg, ip	SA	↓ cue-induced reinstatement (more in STs than in GTs)	[69]
Swiss mice ♂	METH 2 mg/kg, ip	0.5, 2.5 μg/μL, icv	locomotor activity test	↓ METH-induced hyperactivity	[79]
Swiss mice ♂	METH 2 mg/kg, ip	0.5, 2.5 μg/μL into mPFC 2.5 μg/μL into DHC	CPP	↓ stress-reinstained METH-induced CPP	[72]
Long–Evans rats ♀	METH 0.06 mg/kg/infusion, iv; PR	0.3 mg/kg, ip	SA	↓ BP in individually- and socially-housed rats	[81]
Sprague Dawley rats ♂	METH 2 mg/kg, ip	2 mg/kg, ip	locomotor activity test	↓ METH-induced hyperactivity	[82]
Sprague Dawley rats ♂	METH 0.1 mg/kg/infusion, iv 1 mg/kg, ip for METH-priming	1.5, 4.5 pmol (500 nL/side) into NAc core	SA	↓ METH-primed reinstatement	[62]
Sprague Dawley rats ♂	METH 0.1 mg/kg/infusion, iv 1 mg/kg, ip for METH-priming	3.6 pmol (200 nL/side) into the STh	SA	↓ METH-primed reinstatement	[61]
Sprague Dawley rats ♀	METH 0.01, 0.03, 0.1, 0.3, 1 mg/kg, iv 1 mg/kg, ip for METH-priming	1 mg/kg, ip (during adolescence)	SA	↓ METH (0.03 mg/kg) self-administration (in PR and not FR); ↓ METH (1 mg/kg)-primed reinstatement	[44]
Sprague Dawley rats ♂	METH 0.1 mg/kg/50 μL/infusion, iv 1 mg/kg, ip for METH-priming	0.1 μg/side into the PrL	SA	↓ cue-induced METH reinstatement	[68]
		1.0, 3.0 μg/side into the PrL	SA	↓ METH-primed reinstatement	
Sprague Dawley rats ♂	METH 1 mg/kg, ip	0.6 mg, ip	CPP	↓ METH-induced CPP	[38]
		0.6 ng into the NAc core (0.5 μL/side) or into the STh (0.3 μL/side)	CPP		
Sprague Dawley rats ♂, ♀(with SDV)	METH 0.1 mg/kg/50 μL/infusion, iv 1 mg/kg, ip for METH-priming	0.3, 1.0 mg/kg, ip	SA	↓ METH intake in ♂ and ♀ (with SDV-prevention of this OT-induced suppressant effect); ↓ cue- and METH-primed reinstatement (with SDV-prevention of this OT-induced suppressant effect; only in ♂)	[45]
Sprague Dawley rats ♂	METH 0.1 mg/kg, PR, iv 1 mg/kg, ip for METH-priming	0.001, 0.01, 0.1, 0.3 and 1 mg/kg, ip, ascending (prior to self administration) or 1 mg/kg, ip (for reinstatement)	SA	↓ METH intake; ↓ METH-induced hyperactivity; ↓ relapse to METH-seeking behavior	[43]
Sprague Dawley rats ♂, ♀	METH (♀) 17.5 μg/50 μL/infusion, iv (♂) 20 μg/50 μL/infusion, iv	1 mg/kg, ip	SA	↓ cue-induced METH seeking in ♂ and ♀	[63]
		0.6 nmol/0.25 μL/side into NaC core	SA		
C57BL/6 mice ♂, ♀	METH 2 mg/kg, ip	1.25 or 2.5 μg into hippocampus	CPP	↓ context- and restraint stress-induced reinstatement of METH-CPP	[64]
Sprague Dawley rats ♂, ♀	METH (♀) 17.5 μg/50 μL/infusion, iv (♂) 20 μg/50 μL/infusion, iv	1 mg/kg ip	SA (with BE procedure)	↓ METH-demand and ↓ reinstatement to METH-seeking in ♂ and ♀	[66]
		0.6 μg/μL into NAc core	SA (with BE procedure)	↓ METH-seeking	
C57BL/6 mice ♂	METH 2.0 mg/kg, ip	2.5 μg, icv	MWM; NOR	↓ METH-induced spatial memory enhancement ↓ METH-induced cognitive memory deficits	[83]
Sprague Dawley rats ♂, ♀	METH 0.1 mg/kg/50 μL/infusion, iv (followed by ShA or LgA sessions) 1 mg/kg, ip for METH-priming	1 mg/kg, ip (during METH abstinence)	SA EPM	↓ incubation and METH-primed reinstatement in ♂ and ♀ ↓ of LgA-induced heightened anxiety phenotype effects	[70]
Sprague Dawley rats ♂	METH 0.02 ug/50 μL/infusion, iv	1 mg/kg, ip, before reinstatement	SA in rats pre-exposed to a predator odor threat (TMT)	↓ METH-seeking in both saline- and TMT pre-exposed rats	[71]
		1 mg/kg, ip, prior to METH self-administration		↓ stress-induced exacerbation of drug-seeking in TMT pre-exposed rats	
Long–Evans rats ♂ and/or ♀	METH (♀) 17.5 μg/50 μL/infusion, iv (♂) 20 μg/50 μL/infusion, iv 1 mg/kg, ip for METH-priming	1 mg/kg, ip	SA	↓ METH seeking and ↓ PR responding for METH in ♀; ↓ cue-induced METH-reinstatement in ♀; ↓ METH-primed induced METH-seeking in ♂ and ♀	[65]
Sprague Dawley rats ♂	METH 0.1 mg /kg/50 μL/infusion, iv 1 mg/kg, ip for METH-priming	1 mg/kg, ip	SA	↓ METH-primed reinstatement	[67]
		3 pmol (500 nL/side) into the NAcc	SA		
Swiss mice ♂	METH 2 mg/kg, ip	0.1, 0.5, 2.5 μg/μL, icv	CPP	↓ acquisition METH-CPP; facilitation of the extinction of METH-CPP; ↓ restraint stress-induced reinstatement to METH-CPP	[39]
Sprague Dawley rats ♂ (adolescent)	nicotine 25 μg/mL per bottle	0.01 mg/kg, sc	two bottle free-choice paradigm	↓ nicotine aversion after acclimation to nicotine solution	[78]
		1 mg/kg, sc	two bottle free-choice paradigm	↑ nicotine intake	
Wistar rats ♂	nicotine 3.2 mg/kg/day, sc, in osmotic minipump	0.06, 0.125, 0.25, 0.50, 0.75, or 1.0 mg/kg, ip	ICSS and somatic signs evaluation	↓ withdrawal-induced elevations in somatic signs in nicotine-dependent rats with no effect on nicotine withdrawal-induced elevations in ICSS thresholds	[84]
Wistar rats ♂	morphine 5 mg/kg, sc	0.2 μg, icv	CPP	↑ expression, but not acquisition, of morphine-induced CPP	[40]
Wistar rats ♂	morphine 5 and 10 μg/site into the mPFC	5 and 10 ng/site into the mPFC	MWM	↓ morphine-induced decrease in memory related activities	[85]
Sprague Dawley rats ♂	ethanol 10% and 15%	0.1, 0.3, and 0.5mg/kg, ip	three-bottle choice (modified DID model)	↓ ethanol consumption	[48]
	ethanol 10% with gelatin	0.3 mg/kg, ip	SA (oral)		
C57BL/6J mice ♂, ♀	ethanol 12%, 20 μL into the well	(a) 0.5, 1 mg/kg, ip (♀) and 1 mg/kg, ip (♂) (b) 1 mg/kg, ip (♂ and ♀)	SA (oral)	(a) ↓ of TMT-induced reinstatement of ethanol-seeking behavior; (b) ↓ of yohimbine-induced reinstatement of ethanol-seeking behavior	[74]
Wistar rats ♂	ethanol 20%	1 µg/5 µL, icv	two-bottle free-choice paradigm	↓ ethanol consumption	[50]
C57BL/6J mice♂, ♀	ethanol 3 and 6%	3 mg/kg, ip	two-bottle free-choice paradigm with RFIDs	↓ ethanol consumption on 3 out 4 treatment days	[51]
Wistar rats ♂	ethanol 10%, 0.1 mL followed by exposition to ethanol vapor	0.25, 0.5, and 1 mg/kg for FR; 0.125, and 0.25 mg/kg for PR	SA (oral) and alcohol vapor exposure	↓ escalation of ethanol drinking (FR) ↓ enhanced motivation for ethanol (PR)	[52]
		0.25, 0.5 and 1 mg/kg/20 μL; intranasal for FR and 1 mg/kg/20 μL intranasal for PR	SA (oral) and ethanol vapor exposure		
		3, 10 and 30 μg, icv	SA (oral) and ethanolapor exposure	↓ ethanol consumption in dependent rats	
Sprague Dawley rats ♂	ethanol 20%	1 mg/kg, ip	SA (oral)	↓ of yohimbine-induced reinstatement of ethanol-seeking behavior	[73]
		0.5 μg intra-CeA	SA (oral)		
Prairie voles ♂, ♀	ethanol 15%	(a) 1, 3 and 10 mg/kg, ip (b) 3 mg/kg, ip	two-bottle free-choiceparadigm	(a) ↓ ethanol consumption (with restricted access to 15% ethanol) (b) ↓ ethanol consumption (with continuous access to 15% ethanol), depending on time of testing	[54]
Wistar rats ♂	ethanol 15%, 1.5 g/kg, ip	1 µg /5 μL, icv	OF; wire-hanging test;righting-reflex test	↓ ethanol-induced motor impairment (sedation and ataxia)	[86]
C57BL/6N mice ♂	ethanol2, 4, 6 and 8% (escalating)	10 mg/kg, ip	two-bottle free-choice paradigm	↓ ethanol consumption in control but not in CSC-triggered stressed mice	[49]
C57BL/6N mice ♂	ethanol 20%	1, 3, or 10 mg/kg, ip	binge-like DID	↓ ethanol consumption	[46]
		1 mg/kg, ip	two-bottle free-choice paradigm	↓ ethanol consumption	
	ethanol 12%, 20 μL to the well	0.1, 0.3, or 1 mg/kg, ip (FR) and 0.3 mg/kg, ip (PR)	SA (oral)	↓ ethanol consumption (FR) and ↓ motivation to seek ethanol reinforcement (PR)	
Oxt-IRES-Cre mice without viral infusion ♂	ethanol 20%	1 mg/kg, ip	binge-like drinking (DID)	↓ ethanol consumption	[47]
OF1 mice ♂	ethanol 7.6%, 36 μLper nose poke	1 mg/kg, ip	SA (oral)	↓ (social-defeat)-induced increase in ethanol consumption	[53]

BE—behavioral-economic; BP—breaking point (highest number of responses after 4 h or if 1 h elapsed without an infusion); CeA—central amygdala; CPP—conditioned place preference; CSC—subordinate colony housing (for stress induction); DHC—dorsal hippocampus; DID—drinking-in-the-dark model; EPM—elevated plus maze; FR—fixed ratio; GTs—“goal trackers”—rats for whom food reward-paired cue do not become imbued with incentive value, and do not motivate approach behavior; ICSS—intracranial self-stimulation; icv—intracerebroventricular injection; ip—intraperitoneal injection; LgA—long-access (6 h/day); METH—methamphetamine; mPFC—medial prefrontal cortex; MWM—Morris water maze; NAc—nucleus accumbens; NOR—novel object recognition; OF—open field test; PR—progressive ratio; RFIDs—radiofrequency identification tags (for monitoring of individual consumption); SA—self-administration; sc—subcutaneous injection; SDV—subdiaphragmatic vagotomy; ShA—short-access (2 h/day); SI—social interactions paradigm; STh—subthalamic nucleus; STs—“sign trackers”—rats for whom food reward-paired cue acquires control over motivated behaviors; TMT—2,5-dihydro-2,4,5-trimethylthiazoline (predator odor for stress induction); **↓**—decrease; **↑**—increase; ♀—female; ♂—male.

**Table 2:** The effects of OTRs ligands on behavioral/rewarding effects of different drugs of abuse.

Species and Sex	Drug of Abuse and Dose	OTR Ligand and Dose	Behavioral Test	OTR Ligand-Induced Effect	Ref.
Sprague Dawley rats ♂	cocaine 0.25 mg/0.1 mL/infusion, iv	Thr4,Gly7-oxytocin 10 nmol/10 μL, icv	SA	↓ reinstatement of cue-induced cocaine seeking behavior	[60]
Wistar rats ♂	ethanol 10%, 0.1 mL followed by exposition to ethanol vapor	PF-06655075 30 μg, icv	SA (oral) and ethanol vapor exposure	↓ ethanol drinking in dependent rats	[52]
Sprague Dawley rats ♂, ♀	ethanol adolescent intermittent exposure—4 g/kg every 48 h for a total of 11 exposure	WAY-267464 5 mg/kg, ip	SI	reversal of ethanol-induced social anxiety in ♂	[87]
C57BL/6J mice ♂	morphine 20–100 mg/kg/day—escalating, ip	carbetocin 6.4 mg/kg, ip	EPM, FST, sociability and social novelty test	↓ withdrawal-induced negative emotional consequences (↓ of anxiety- and depressive-like and restoration of sociability behaviors)	[75]
	morphine 10 mg/kg, sc	carbetocin 6.4 mg/kg, ip	CPP	prevention of stress-induced reinstatement to morphine-seeking	
C57BL/6J mice ♂	morphine 10 mg/kg, sc for conditioning; 2 mg/kg, ip for morphine-priming	carbetocin 6.4 mg/kg, ip	CPP	prevention of morphine priming-induced reinstatement to morphine CPP	[76]
C57BL/6 mice ♂	ethanol 10%, 2 g/kg, ip	carbetocin 6.4 mg/kg, ip	CPP	↓ acquisition of ethanol CPP; facilitation of extinction of ethanol-CPP; ↓ reinstatement induced by ethanol priming	[77]
Sprague Dawley rats♂, ♀	MDMA 1.5 mg/kg, ip amphetamine 1 mg/kg, ip	carbetocin 2 and 20 mg/kg, ip	three-lever drug discrimination paradigm (MDMA/ amphetamine/saline)	↑ MDMA lever presses—carbetocin generalized to the MDMA training cue; no effect on amphetamine lever selection	[88]
Swiss mice ♂	ethanol 20%, 2 g/kg, ip	carbetocin 6.4 mg/kg, ip	CPP	mimicking of behavioral effects of EE on ethanol-CPP ↑ of ethanol-CPP	[89]
		L,368,899 5 mg/kg, ip (during EE exposure but not during acquisition of ethanol CPP)	CPP	↓ EE-induced ethanol CPP	
C57BL/6J mice ♂	MDMA 3 mg/kg, ip	L,368,899 10 mg/kg, ip	sociability test	↓ prosocial effects of MDMA in highly sociable mice; no effect in low sociable mice	[90]
Oxt-IRES-Cre mice with intra-PVN infusion of active virus * ♂	ethanol 20%	L,368,899 10 mg/kg, ip	binge-like drinking (DID)	reversal of (chemogenetic activation of PVN OT neurons)-induced ↓ of binge-like ethanol drinking	[47]
C57BL/6J mice ♂	ethanol 20%	L,368,899 10 mg/kg, ip	binge-like drinking (DID)	↓ of OT-induced reduction in binge-like ethanol consumption	[46]
Swiss mice ♂	METH 2 mg/kg, ip	atosiban 2 μg/μL, icv	locomotor activity test	↓ inhibitory effect of OT (0.5, 2.5 μg) on METH-induced hyperactivity in mice	[79]
Swiss mice ♂	METH 2 mg/kg, ip	atosiban 10 μg/μL into mPFC	CPP	↓ OT (2.5μg/μL into mPFC)-induced inhibition of stress-reinstained METH-induced CPP	[72]
Swiss mice ♂	METH 2 mg/kg, ip	atosiban 2.0 μg/μL, icv	CPP	↓ OT-induced effects (see Table 1)	[39]
OF1 mice ♂	cocaine 1 mg/kg, ip	atosiban 1 mg/kg, ip	CPP	reversal of (positive social housing)-protective effect against increased cocaine reward	[91]
Sprague Dawley rats ♂, ♀	MDMA 1.5 mg/kg, ip amphetamine 1 mg/kg, ip	atosiban 10 mg/kg, ip	three-lever drug discrimination paradigm (MDMA/ amphetamine/ saline)	disruption of MDMA- (but not AMP-) appropriate responding	[88]
Wistar rats ♂	MDMA 5 mg/kg, ip	tocinoic acid 20 μg/μL, icv	SI	↓ the facilitation of MDMA-induced social interactions	[92]

* with chemogenetic activation of OT-containing neurons; CPP—conditioned place preference; DID—drinking-in-the-dark model; EE—model of “eustress”—animals were exposed to different stimuli, such as toys, tubes, ladders, houses and running wheels (objects were changed/moved three times a week); EPM—elevated plus maze; FST—forced swim test; HPA—hypothalamic-pituitary-adrenal; icv—intracerebroventricular injection; ip—intraperitoneal injection; METH—methamphetamine; OT analog—Thr4,Gly7-oxytocin; OTRs agonists—PF-06655075, WAY-267464, carbetocin; OTRs antagonists—L,368,899, atosiban, tocinoic acid; SA—self-administration; sc—subcutaneous injection; SI—social interactions paradigm; ↓—decrease; ↑—increase; ♀—female; ♂—male.

### 3.2. The Involvement of AVP Transmission in Drug-Induced Reward

Research suggests that the administration of AVP (similarly to the administration of OT) can lead to the amelioration of drug-induced reward; however, the amount of available data is less abundant than reported for OT (Table 3). Specifically, it has been shown that central administration of AVP into the lateral septum (LS) blocked the expression of amphetamine-induced CPP [93]. Interestingly, the effects of the AVPV_1A_Rs antagonist (SR49059) and the AVPV_1B_Rs antagonist (SSR149415) on the drug-induced reward in rodents seem to be opposite (Table 4). Precisely, the effects of the AVPV1ARs blockage by its antagonist, SR49059, prevented OT-induced attenuation of METH-primed reinstatement in rats [67]. Conversely, the effects of the AVPV_1B_Rs blockage by its antagonist, SSR149415, seem to be opposite to the effects of the AVPV_1A_Rs blockage. Specifically, the administration of SSR149415 resulted in a decrease in ethanol intake [57,94,95], blockage of the acquisition of morphine-CPP in rats housed with identically treated conspecifics [14], attenuation of foot shock- and heroin priming-induced reinstatement to heroin self-administration [96] and prevention of nicotine withdrawal-induced dysphoria in intracranial self-stimulation (ICSS) [97]. The above-mentioned AVPV_1A_Rs and AVPV_1B_Rs distinctions suggest that a drug-induced reward in rodents may be attenuated by the blockage of AVPV_1B_Rs, whereas the blockage of AVPV_1A_Rs prevents the amelioration of a drug reward. Interestingly, the SR49059-induced AVPV1ARs blockage prevented the acquisition of MDMA-CPP in adult zebrafish [98], indicating a possible species-dependent effect; however, available data are insufficient to fully confirm this hypothesis. The effects of AVP and AVPRs ligands on behavioral/rewarding effects of different drugs of abuse has been summarized in Table 3 and Table 4, respectively. 

**Table 3:** The effects of AVP on behavioral/rewarding effects of different drugs of abuse.

Species and Sex	Drug of Abuse and Dose	AVP Effective Dose	Test	AVP-Induced Effect	Ref.
Long–Evans rats ♂	MDMA	0.0025 mg/kg, ip	SI	↑ adjacent lying	[80]
	2.5 mg/kg, ip				
Wistar or Sprague Dawley rats ♂	ethanol 4.5% (or CIE or modified CIE)	4 μg or 0.4 μg/0.5 μL * into CEA	SI	↑ ethanol withdrawal-induced social anxiety	[99]
Rats **	amphetamine	50 ng, icv	normal/abnormal behavior assessment ***	cross-sensitization of rats to amphetamine hyperlocomotion	[100]
Sprague Dawley rats ♂	amphetamine	0.2 ng/side into LS	CPP	↓ expression of amphetamine-induced CPP	[93]

* 4 μg/0.5 μL in both strains and 0.4 μg/0.5 μL only in Wistar rats; ** only information provided—Charles River strain; *** abnormal behaviors associated with AVP treatment: severe motor disturbances, sprawled-out posture with hind-limb extension and motor difficulty, and excessive scratching; abnormal behaviors associated with amphetamine administration: excessive locomotion and motor stereotypy; CEA—central nucleus of the amygdala; CIE—chronic intermittent ethanol exposure; CPP—conditioned place preference; icv—intracerebroventricular injection; ip—intraperitoneal injection; LS—lateral septum; SI—social interactions paradigm; ↓—decrease; ↑—increase; ♂—male.

**Table 4:** The effects of AVPRs ligands on behavioral/rewarding effects of different drugs of abuse.

Species and Sex	Drug of Abuse and Dose	AVPR Ligand and Dose	Test	AVPR Ligand-Induced Effect	Ref.
adult zebrafish	MDMA 5 mg/kg im	SR49059 0.01, and 0.1 ng/kg, im	CPP	↓ MDMA-induced CPP	[98]
		SR49059 0.01, and 0.1 ng/kg, im	social preference test	↓ MDMA-induced social preference	
	MDMA 10 mg/kg im	SR49059 0.1 and 1 ng/kg, im	novel tank diving test	↓ MDMA-induced anxiolytic effect	
	MDMA 2.5 mg/kg im	SR49059 0.01 and 0.1 ng/kg, im	light-dark test	↓ MDMA-induced anxiolytic effect	
Sprague Dawley rats ♂	METH 0.1 mg/kg/50 μL/ infusion, iv; 1 mg/kg, ip for METH-primed reinstatement	SR49059 1 mg/kg, ip	SA	↓ OT-induced prevention of METH-primed reinstatement	[67]
Long–Evans rats ♂	MDMA 5 mg/kg, ip	SR49059 1 mg/kg, ip	SI	↓ MDMA-induced adjacent lying	[80]
Sprague Dawley rats ♂, ♀	ethanol adolescent intermittent exposure—4 g/kg every 48 h for a total of 11 exposures	SSR149415 5, 10, 20 mg/kg, ip	SI	↓ ethanol-induced social anxiety	[87]
Wistar rats ♂	nicotine 0.4 mg/kg, sc	SSR149415 30 mg/kg, ip	locomotor activity test	↓ expression of nicotine-induced sensitization	[101]
C57BL/6J mice ♂, ♀	ethanol 15%	SSR149415 10 and 30 mg/kg ip	two-bottle choice paradigm with IA	↓ ethanol intake and preference	[57]
		SSR149415 1 and 3 mg/kg + naltrexone 1 mg/kg			
sP and sNP rats ♂	ethanol 10%	SSR149415 30 mg/kg, ip	two-bottle choice paradigm	↓ ethanol intake in sP rats	[95]
Wistar rats ♂	nicotine 3.16 mg/kg/day in osmotic sc minipumps	SSR149415 0.1, 0.5, and 2 μg, icv (acute); 0.5 μg/day for 6 days, icv (chronic)	ICSS (mecamylamine-precipitated nicotine withdrawal)	*chronic treatment:* complete prevention of the elevations in brain reward thresholds linked with nicotine withdrawal (prevention of the nicotine withdrawal-caused dysphoria); *acute treatment*: partial prevention of nicotine withdrawal	[97]
C57BL/6N mice ♂	morphine 10, 20 or 40 mg, sc (6 day progressive ratio)	SSR149415 10 mg/kg, ip	CPP	↓ acquisition of morphine-CPP in the morphine only mice * (no effect on the acquisition of morphine CPP in the morphine cage-mate mice **)	[14]
Wistar rats ♂	ethanol CIEV adjusted by controlling BALs	SSR149415 30 mg/kg, ip	SA	↓ excessive levels of ethanol SA observed in dependent animals without affecting ethanol drinking in non-dependent animals	[94]
Fischer rats ♂	heroin 0.05 mg/kg/infusion, iv 0.25 mg/kg, sc, for priming	SSR149415 30 mg/kg, ip	SA	↓ foot shock-induced heroin reinstatement ↓ heroin-primed heroin reinstatement	[96]
Fischer rats ♂	cocaine 45–90 mg/kg/day, ip (chronic binge pattern with EDC)	SSR149415 5 mg/kg, ip	chronic EDC binge cocaine with acute withdrawal paradigm	↓ acute withdrawal-induced HPA axis activation (ACTH increase) after EDC	[102]
Wistar rats ♂	ethanol 4.5% modified CIE or CIE	SSR149415 5 μg in 0.5 μL into CEA	SI	binary effect: ↓ of social interactions in control animals but reversal of ethanol withdrawal-induced decrease in social interactions	[99]

* morphine-treated animals housed with only morphine-treated animals; ** morphine-treated animals housed with drug-naïve anim; BALs—blood alcohol levels; CEA—central nucleus of the amygdala; CIE—chronic intermittent ethanol exposure; CIEV—chronic, intermittent, exposure to ethanol vapors; CPP—conditioned place preference; EDC—escalating-dose cocaine; IA—chronic intermittent access; icv—intracerebroventricular injection; ip—intraperitoneal injection; SA—self-administration; sc—subcutaneous injection; SI—social interactions paradigm; sNP—Sardinian alcohol-nonpreferring rats; sP—Sardinian alcohol-preferring rats; SR49059—AVPV_1A_Rs antagonist; SSR149415—AVPV_1B_Rs antagonist ↓—decrease; ♀—female; ♂—male.

### 3.3. The Relationship between Social Factor and OT/AVP Impact on the Effects of Drugs of Abuse

Data summarized in the previous sections (Section 3.1 and Section 3.2) undeniably show that alternations in OT and AVP transmission may influence the drug-induced reward. Nevertheless, those effects can be additionally strongly modified by a social factor.

In rats, it has been shown that MDMA, OT and AVP do not induce classic-CPP but, interestingly, MDMA and OT are able to produce social-CPP (conducted with the presence of another conspecific during conditioning sessions) [3]. In the social interactions test MDMA, OT and AVP increased the time of the adjacent lying of rats (a prosocial effect) and this effect was attenuated by SR49059 (AVPV_1A_Rs antagonist), suggesting an involvement of these receptors in the observed phenomenon [80]. A similar pattern has been reported in zebrafish, where SR49059 was able to block MDMA-induced CPP and decrease MDMA-induced social preference [98]. In terms of OTRs, it has been shown that the OTR agonist, tocinoid acid, was able to attenuate the MDMA-induced facilitation of social interactions in rats [92] and that the OTR antagonist, L-368,899 was able to abolish the prosocial effects of MDMA in highly sociable mice with no effect on low-sociable mice [90]. Conversely, another OTRs antagonist, compound **25** (5-{3-[3-(2-chloro-4-fluorophenoxy)azetidin-1-yl]-5-(methoxymethyl)-4H-1,2,4-triazol-4-yl}-2-methoxypyridine), failed to attenuate an MDMA-, OT- and AVP-induced increase in adjacent lying [80], pointing to the need for further clarification. What needs to be highlighted is that MDMA is an empathogenic/entactogenic drug with strong prosocial effects in humans [103,104]. This is a distinctive feature that differentiates this drug from other psychoactive drugs reviewed in this paper. Nevertheless, the social factor has also been shown to alter OT-/AVP-dependent rewarding effects of other drugs of abuse.

**Figure 3:**
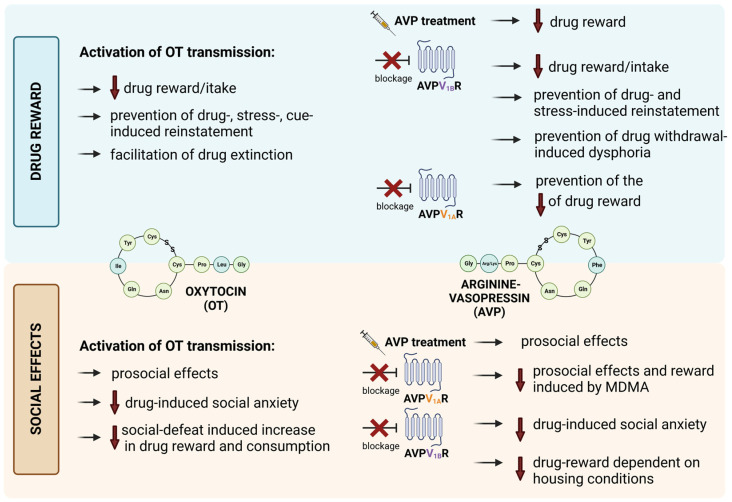
The summary of available data on OT/AVP effects on drug reward and social effects. Detailed descriptions are presented in Section 3.1, Section 3.2 and Section 3.3. This figure was created with https://biorender.com/ (accessed on 10 January 2023).

It has been reported that OT can attenuate the social defeat-induced increase in cocaine reward and attenuate the cocaine-primed reinstatement of social defeat-induced cocaine seeking [59]. OT also decreased the social defeat-induced increase in ethanol consumption [53] and the OTR agonist (WAY-267464) has been shown to reverse ethanol-induced social anxiety [87]. Treatment with the AVPV_1B_Rs antagonist (SSR149415) also led to attenuation of ethanol-induced social anxiety [87]. SSR149415 also produced an interesting effect in the morphine-CPP experiment where it was able to block the acquisition of morphine-CPP in morphine-treated animals housed with only morphine-treated animals, but had no effect on morphine-CPP in morphine-treated animals housed with drug-naïve animals [14]. The graphical summary of the OT/AVP-dependent social effects is presented in Figure 3.

All of the above-mentioned examples show that the social factor is a highly important component of OT/AVP-dependent drug reward. Unfortunately, because the vast majority of currently available studies did not take this variable into consideration, full understanding of this relationship is yet to be discovered. Nevertheless, some hypotheses can be proposed.

Firstly, most studies in this area have been performed on rodents which are naturally social animals [15] in which negative social encounters can facilitate the development of drug addiction [105] and in which drug withdrawal can lead to social impairment [106]. Conversely, positive social interactions and social experience have the potential to manage or overcome drug addiction, most likely due to the backward shift of the drug reward onto social reward. In the light of this hypothesis (excellently reviewed in [93]), OT is able to: (a) reduce drug reward by decreasing the DA release and DA transmission in brain areas involved in drug addiction [38,39,50,66,79,107]; (b) enhance social reward by increasing positive prosocial behaviors [80]) and by restoring drugs-induced social deficits [75,108,109]; (c) regulate emotional states by decreasing drugs- and cue-induced anxiety [60,70,75] and drugs-induced depressive-like behaviors [75].

Secondly, following a closer look into the OT/AVP-related effects in different drugs of abuse (Table 1, Table 2, Table 3 and Table 4), a specific pattern can be observed, suggesting that these OT/AVP effects may be dependent on the type of the studied drug (empathogens vs. other psychoactive drugs). As mentioned above, MDMA is a classic empathogenic drug that in humans increases empathy, willingness to socialize and being close to others, talkativeness, amicability, and gregariousness [103]. In animal models, MDMA, OT and AVP triggered similar prosocial effects (increase in adjacent lying, induction of social- but not classic-CPP) [3,80]. Additionally, MDMA administration has been reported to increase plasma OT level in animals [92] and humans [110,111]. Conversely, numerous available studies on various psychoactive drugs that are not classified as empathogens (e.i. morphine, cocaine, ethanol) showed that chronic exposure to these drugs leads to a reduction in the plasma and central OT level [75,76,112,113]. Interestingly, in rats trained to discriminate MDMA, amphetamine and saline in a three-lever drug discrimination paradigm, carbetocin (an OTRs agonist) substitution led to an increase in MDMA-lever presses when compared to saline-lever presses while atosiban (an OTRs antagonist) was able to selectively disrupt MDMA-, but not amphetamine-, lever responding [88]. This distinction between MDMA and other mentioned drugs may indicate that OT/AVP-related effects are different in empathogens and classic psychostimulants. This observation needs to be taken into consideration in future studies on new empathogenic drugs.

Thirdly, an important aspect of an unaware social component in the experiment design needs to be discussed. Although many studies on OT/AVP involvement in drug reward did not take into consideration the impact of social factors on their results, an unintended and unplanned effect of the social variables, such as (a) housing; (b) same/different treatment regimen with/without cage mates and (c) waiting time between drug administration and testing with/without other conspecifics in home cages/transfer cages; are additionally important factors that can influence the experience derived from drug administration. This is specifically important for experiment designs that involve treatment with OT/AVP or their ligands. As mentioned above, it has been already proven that housing conditions (housing with drug- or vehicle-treated cage mates) can affect the acquisition of morphine-CPP [14]. Therefore, based on the above-mentioned reasons, a strong conclusion should be stated that while studying drug- and social-reward, as well as the OT/AVP-dependent effects of addictive substances, a social factor should be considered as an important variable and should not be neglected in the experiment design and analysis.

### 3.4. The Impact of OT/AVP and OTRs/AVPRs Ligands on Other Behavioral Effects of Drugs of Abuse

Although the main objective of this review is to elaborate on the interaction between OT/AVP and drug-reward, the impact of these neuropeptides on other drug-induced behavioral effects cannot remain unremarked on. Specifically, the OT/AVP impact on anxiety- or depressive-like behaviors (as indicators of emotional states), memory performance and locomotor effects needs to be highlighted.

Available research reported that OT may decrease anxiety (measured in an elevated plus maze (EPM) test) triggered by long access (LgA) to METH [70] and by the reinstatement induced by cocaine and cues [60]. A similar effect has been observed for the OTRs agonist, carbetocin, which was able to reduce morphine withdrawal-induced anxiety and depressive-like behaviors evaluated in the EPM and forced swim test (FST), respectively [75]. In terms of memory performance, OT was able to decrease METH-induced cognitive memory deficits in the novel object recognition (NOR) test [83] and to decrease morphine-induced spatial memory deterioration in the Morris water maze (MWM) test [85]. However, OT also decreased METH-induced spatial memory enhancement in the MWM [83], suggesting drug- and memory-type-dependent effects. OT also reduced METH-induced hyperlocomotion [79,82] and the OTRs antagonist, atosiban, was able to block this OT-induced effect [79]. Furthermore, OT prevented ethanol-induced motor impairment assessed in an open field (OF), wire-hanging and righting-reflex test [86]. Finally, OT was able to reduce withdrawal somatic signs in nicotine-dependent animals [84].

The effects of AVP treatment on anxiety levels are difficult to assess due to the scarcity of available data. Nevertheless, it has been reported that AVP microinjected into the central nucleus of the amygdala (CEA) can induce social anxiety-like behavior in rats exposed to chronic ethanol and that SSR149415 (AVPV_1B_Rs antagonist) was able to reduce this ethanol-induced social anxiety [99]. Interestingly, administration of SR49059 (AVPV_1A_Rs antagonist) in zebrafish led to a decrease in the MDMA-induced anxiolytic effect [98], which further supports the hypothesis of the bidirectional effects of AVPV_1A_Rs and AVPV_1B_Rs antagonists. In terms of AVP effects on animals’ locomotion, it has been shown that AVP treatment leads to cross-sensitization to amphetamine-induced hyperlocomotion [100] and that SSR149415 is able to decrease the expression of nicotine-induced locomotor sensitization [101]. The impact of AVP and AVPRs ligands on depressive-like behavior and memory performance is yet to be discovered.

## 4. Interactions between OT/AVP Transmission, DA Release and Drug Reward System

Ample evidence has proven the involvement of OT and AVP in drugs of abuse-related effects, showing that OT/AVP treatment contributes to the attenuation of drug consumption and reward (Table 1, Table 2, Table 3 and Table 4). Therefore, recognizing the interactions between OT/AVP, DA and the drug reward system is crucial to fully understand the mechanisms underlying the observed phenomenon. Although it is a complex and extensive issue, several aspects must be highlighted.

Firstly, OT has been shown to directly interact with dopaminergic brain reward regions. For example, OTRs have been found on the VTA dopaminergic neurons that project from the PVN into the NAc and medial prefrontal cortex (mPFC) [114,115]. The location of OTRs in the mesocorticolimbic structures triggers direct OT and DA interactions. It has been reported that subchronic OT treatment decreased DA release in the NAc and amygdala in drug non-exposed animals [107]. Furthermore, central OT administration blocked DA release in the NAc in ethanol-treated rats [50], inhibited METH-induced DA turnover in the NAc [79] and decreased DA release [38,66] in the NAc. Importantly, chronic treatment with various drugs of abuse has been shown to decrease plasma and the central OT level in animal models [75,76,112,113,116], which suggests drug-induced hypofunction of the OT system and may contribute to the dysfunction of social behavior and further development of addiction [117]. These findings were consistent with effects observed in humans where chronic cocaine use during pregnancy led to a decrease in the plasma OT level and mood disruption in mothers [118].

Another aspect that needs highlighting is a differentiation in the role of dopaminergic D_1_ and D_2_ receptors (D_1_Rs and D_2_Rs, respectively) in addiction. Available reports suggest that D_1_Rs are mainly involved in drug reward whereas D_2_Rs are mostly involved in social reward [106,119]. Drug administration leads to an increased level in DA which can interact with D_1_Rs, as well as D_2_Rs, creating both drug- and social-related rewarding effects, respectively [120]. Nevertheless, chronic drug consumption has been linked with decreased DA levels in the basal striatum and downregulation of D_2_Rs [105,106,120]. This may contribute to higher D_1_Rs versus D_2_Rs activation and further reinforce drug-reward and weaken social-reward [106]. Interestingly, recent studies have reported that OTRs can form heterodimer complexes with D_2_Rs (OTRs/D_2_Rs) in NAc, amygdala and the dorsal striatum where the activation of OTRs led to increased D_2_Rs signaling (by increased affinity and density of D_2_Rs) [121,122,123,124,125]. Therefore, OT may be able to reverse the above-mentioned imbalance in D_1_Rs/D_2_Rs activation, increase D_2_Rs activation and thus, shift the drug-reward into social-reward [106]. Taking into account the fact that drug abuse can lead to social impairment [126], the OT regulating hypothesis is strongly relevant in terms of OT use in the treatment of drug addiction and drug-induced social disruptions.

Regrettably, current knowledge on AVP and DA interactions is scarce. Hitherto, it has been shown that amphetamine treatment leads to a decrease in AVP levels in LS [93,127] and that AVP microinjected into LS decreases DA release in the NAc 930. Furthermore, an increase in striatal expression of D_1_Rs (but not D_2_Rs) and an increase in the striatal AVP expression level was observed in morphine-treated animals when housed with identically treated conspecifics but not when housed with drug-naive animals [14], which highlights the significance of social factors in the development of drug addiction. Interestingly, AVP gene expression analysis showed its down-regulation during early- and mid-acquisition and up-regulation during the late-acquisition and expression of environment-elicited cocaine conditioning [128], which suggests addiction stage-dependent changes. Nevertheless, further studies are needed to fully elucidate the interaction between the AVP and DA mesocorticolimbic system in the context of drug reward.

## 5. Interactions between OT/AVP and Serotonergic Transmission

One of the most important findings in the area of interest has been presented by Dölen et al. (2013) [129], who showed that social reward is dependent on the combined action of OT and serotonin (5-HT) in the NAc core. The activation of OTRs within the NAc of mice (which are colocalized with presynaptic terminals of serotonergic inputs from the dorsal raphe nucleus) leads to the release of 5-HT and is required for the social reinforcement measured in social-CPP. Interestingly, this effect is abolished by the presence of the 5-HT1B antagonist [129]. This finding may be specifically important in terms of elucidating the difference between the social reward of empathogens (such as MDMA) and other psychoactive drugs. The most prominent difference between these drugs is the intensity of interaction with the serotonergic transmission. MDMA (apart from classic dopaminergic mechanisms) can strongly interact with the 5-HT transporter (SERT) and stimulate the release of 5-HT which is (along with OT mechanisms) responsible for the prosocial effects [130]. This combined strong interactions with OT/AVP [3,80,92] and serotonergic transmission [130] seems to be the key feature of MDMA empathogenic and prosocial activity. This may be also connected with the fact that OT infusion is able to promote 5-HT release within the median raphe nucleus which has been linked with subsequent anxiolytic activity [131].

Interestingly, the OT/5-HT interactions have additionally been shown to take part in important aspects of motherhood, such as postpartum nursing, anxiety, aggression and stress management [132]. Several studies have also demonstrated the significance of OT/5-HT interactions in the autistic spectrum disorder (ASD), a condition that is characterized, among other features, by disruptions in social behavior [133,134]. Specifically, OT/5-HT interactions via 5-HT_1A_ receptors have been shown to play an important role in the development of social behavior showing a possible novel strategy for the treatment of ASD [135].

## 6. Clinical Trials of Intranasal OT in Drug Abuse

The importance of conducting further preclinical studies on OT/AVP involvement in drug addiction is supported by promising results of available clinical trials on intranasal OT in the treatment of drug abuse. This route of administration allows us to increase central oxytocin levels through a direct nose-to-brain delivery [136]. Specifically, intranasal OT has been shown to reduce [137] heroin cravings. Additionally, intranasal OT has been reported to improve social perception and decrease the appetitive approach [138], as well as to reduce ethanol cravings and withdrawal symptoms in humans with ethanol abuse disorder [138,139]. Furthermore, intranasal OT has successfully decreased cocaine [140], nicotine [141] and cannabis [142] craving or use, as well as reducing cannabis-induced anxiety [142]. The above-mentioned examples certainly prove the possibility of the utility of intranasal OT in the treatment of drug addiction. However, these positive and promising effects need to be counterbalanced with other reports indicating that intranasal OT does not influence ethanol withdrawal scores [143], may have a negative effect on ethanol-dependent patients with anxious emotional states [138] and increase the craving for cocaine use [144]. Finally, it needs to be mentioned that all of the above-mentioned effects may be dependent on: (a) the administered dose—40 IU [137,138,140,141,142] vs. 24 IU [139,143,144] and (b) dosage regimen, namely single [137,138,141,142] vs. repeated administration [139,140,143]. Therefore, creating an appropriate dosage model may be the key to going successfully through all stages of the clinical trials and drug registration process.

## 7. Conclusions and Future Research Directions

All of the findings reviewed in this paper certainly prove the importance of OT and AVP systems as promising targets for the management of drug addiction. One of the most important observations is that OT has been shown to be able to reverse drug-induced social disruptions and to shift drug-induced reward into social-induced reward. These complementing effects of decreasing drug reward on the one hand, and reducing drug-related social impairment on the other, seem to be a unique feature in currently available pharmacological treatment options for drug abuse. Further, ever more studies in animal models indicate the potential utility of the AVPRs ligands in the management of addiction. However, since the quantity of data is still scarce, future studies should: (a) focus on advancing knowledge about AVPRs ligands, aim to select the most promising substance for possible clinical trials; (b) clarify the bidirectional effects of AVPV_1A_Rs and AVPV_1B_Rs in the mitigation of drug-reward; (c) take social factors into consideration while designing the drug experiment and incorporate it in results analysis; (d) further evaluate binary OT/AVP-dependent effects in empathogens vs. other psychoactive substances—preferably in newly synthesized MDMA-like empathogens. Explanation of these above-mentioned unknown phenomena will undeniably broaden our understanding of the role of OT and AVP in drug abuse and contribute to the development of effective interventions for treating substance use disorders.

## Data Availability

Not applicable.
